# Semi-automated segmentation and quantification of adipose tissue in calf and thigh by MRI: a preliminary study in patients with monogenic metabolic syndrome

**DOI:** 10.1186/1471-2342-6-11

**Published:** 2006-08-31

**Authors:** Salam A Al-Attar, Rebecca L Pollex, John F Robinson, Brooke A Miskie, Rhonda Walcarius, Brian K Rutt, Robert A Hegele

**Affiliations:** 1 Vascular Biology Research Group, Robarts Research Institute, London, Ontario, Canada; 2 Imaging Research Laboratories, Robarts Research Institute, London, Ontario, Canada; 3 Department of Medicine, Schulich School of Medicine and Dentistry, University of Western Ontario, London, Ontario, Canada

## Abstract

**Background:**

With the growing prevalence of obesity and metabolic syndrome, reliable quantitative imaging methods for adipose tissue are required. Monogenic forms of the metabolic syndrome include Dunnigan-variety familial partial lipodystrophy subtypes 2 and 3 (FPLD2 and FPLD3), which are characterized by the loss of subcutaneous fat in the extremities. Through magnetic resonance imaging (MRI) of FPLD patients, we have developed a method of quantifying the core FPLD anthropometric phenotype, namely adipose tissue in the mid-calf and mid-thigh regions.

**Methods:**

Four female subjects, including an FPLD2 subject (*LMNA *R482Q), an FPLD3 subject (*PPARG *F388L), and two control subjects were selected for MRI and analysis. MRI scans of subjects were performed on a 1.5T GE MR Medical system, with 17 transaxial slices comprising a 51 mm section obtained in both the mid-calf and mid-thigh regions. Using ImageJ 1.34 n software, analysis of raw MR images involved the creation of a connectedness map of the subcutaneous adipose tissue contours within the lower limb segment from a user-defined seed point. Quantification of the adipose tissue was then obtained after thresholding the connected map and counting the voxels (volumetric pixels) present within the specified region.

**Results:**

MR images revealed significant differences in the amounts of subcutaneous adipose tissue in lower limb segments of FPLD3 and FPLD2 subjects: respectively, mid-calf, 15.5% and 0%, and mid-thigh, 25.0% and 13.3%. In comparison, old and young healthy controls had values, respectively, of mid-calf, 32.5% and 26.2%, and mid-thigh, 52.2% and 36.1%. The FPLD2 patient had significantly reduced subcutaneous adipose tissue compared to FPLD3 patient.

**Conclusion:**

Thus, semi-automated quantification of adipose tissue of the lower extremity can detect differences between individuals of various lipodystrophy genotypes and represents a potentially useful tool for extended quantitative phenotypic analysis of other genetic metabolic disorders.

## Background

The metabolic syndrome (MetS) related to a pattern of central or abdominal obesity is a major health concern in the westernized world. One approach to begin to understand a common complex trait such as MetS is to closely study individuals who have a rare monogenic analogue of the condition. In the case of MetS, the familial partial lipodystrophy syndromes represent an extreme monogenic model system that demonstrates the salient clinical (increased blood pressure and increased abdominal fat) and biochemical manifestations (increased plasma glucose and triglyceride concentrations and decreased plasma HDL cholesterol concentration).

The two molecular forms of autosomal dominant Dunnigan-type familial partial lipodystrophy (FPLD) result from mutations either in *LMNA *encoding nuclear lamin A/C (FPLD2; MIM 151660) or in *PPARG *encoding peroxisome proliferator-activated receptor-γ (FPLD3; MIM 604367) [1-3]. One in 100,000 individuals has FPLD in North America. Patients with either form of this rare disorder show loss of subcutaneous fat, especially from extremities, together with predisposition to insulin-resistant diabetes, dyslipidemia and hypertension [1-3]. Despite the similar clinical course, there are subtle clinical phenotypic differences between FPLD2 and FPLD3 [1-3]. For instance, compared to FPLD2 subjects, FPLD3 subjects appear to have less severe adipose involvement on physical examination, together with more severe clinical and biochemical manifestations of insulin resistance, and more variable response to treatment with thiazolidinedione drugs [2,3].

To date, thorough semi-quantitative descriptions of the localization and extent of fat loss from affected tissues have taken advantage of both clinical assessment and, more recently, non-invasive imaging methods, such as magnetic resonance imaging (MRI) [4-6]. While the descriptions of MR images in FPLD patients have been extensive, thorough and detailed, they have not yet been quantitative [4-6]. Because quantitation of fat mass on MRI could: 1) enhance the description of these rare disorders; 2) allow for statistical comparisons; and 3) yield new quantitative traits to follow serially, it is important to develop robust and replicable tools and methods to quantify subcutaneous fat [7,8]. We now report a method to quantify lower extremity fat depots in patients with FPLD2 and FPLD3 from serial MR images that utilized an almost completely automated strategy. Using the method developed, the study further reports on quantitative differences between lower extremity adipose tissue distribution in the case of two FPLD patients along with comparisons to matched controls.

## Methods

### Study subjects

All study subjects were female. The study sample included an FPLD2 subject (designated GL0096) and an FPLD3 subject (designated GL0658), a young control subject (designated GL2784) whose body mass index (BMI) was matched to the FPLD2 subject and an older control subject (designated GL2990) whose age was matched with both FPLD subjects and whose BMI was matched to the FPLD3 subject (Table 1). All subjects provided informed consent to participate and human ethics approval was obtained from the University of Western Ontario Institutional Review Board (protocol #11244).

### Clinical and biochemical assessment

All subjects provided a medical history and were subjected to a complete physical examination. Bioimpedance analysis (BIA) measurements were also gathered using the Tanita BC-418 Segmental Body Composition Analyzer (Tanita, Arlington Heights, IL) providing estimates of percent fat for the total body and lower right and left extremities. The average of three measurements was reported for each BIA value.

### Magnetic resonance imaging and image analysis

MRI scans were obtained at the London Health Sciences Centre, University Campus, London, Ontario. Scanning was performed on a 1.5T GE MR Medical system (Model: Signa Excite) using an 8-channel receive-only torso array coil. Images of the various sections were acquired using a T1-weighted Spin Echo pulse sequence with the following parameters: FOV of 40 cm for mid-calves and 48 cm for mid-thighs, TR/TE 400/10 ms, bandwidth +/-15.63 kHz, 2 NEX (number of signal averages), and an acquisition matrix of 256 × 256.

**Figure 1:**
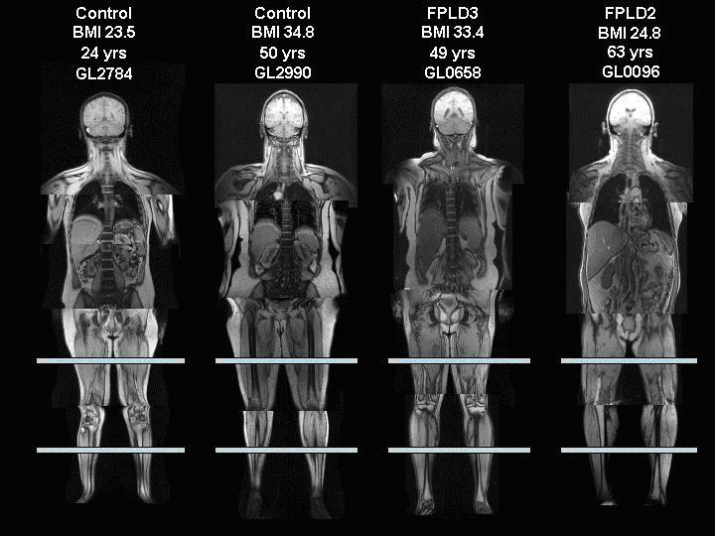
**Full body coronal magnetic resonance image of the subjects in the study and the assigned survey fields in the mid-calf and mid-thigh**. On these survey images the horizontal bars indicate the location of the mid-calf and mid-thigh sections, positioned based on reference anatomical features. The mid-point of the tibia was selected for "mid-calf" measurements and the mid-point of the femur was selected for "mid-thigh" measurements. Subjects from left to right are normal controls, GL2784 and GL2990, followed by the FPLD3 patient (GL0658) and FPLD2 patient (GL0096).

**Figure 2:**
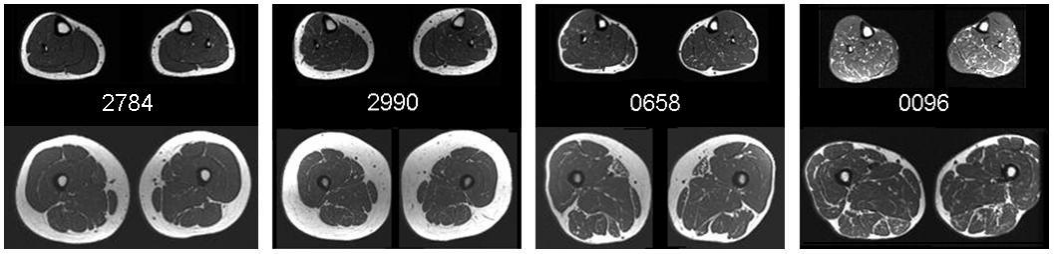
**Transaxial magnetic resonance images at the levels of mid-calf (top slice images) and mid-thigh (bottom slice images) of the subjects in the study**. Bright/white signals in these images are highlighting adipose tissue within these anatomical sections. Dark signals represent either muscle tissue within sections or the background of the images. Subject GL2784 is a healthy 24 year old woman whose MRI showed no infiltrated fat into calf muscle, and only small amount of infiltration in the thigh. Subject GL2990 is a normal 50 year old woman who had somewhat increased subcutaneous (sc) fat in the calves and mid-thigh with slightly more infiltration of fat into the muscle compared to the images of subject GL2784. Subject GL0658 is a 49 year old FPLD3 patient (heterozygous for mutation *PPARG *F388L) whose scans show moderate loss of sc fat in both the calves and mid thigh and moderate levels of fat infiltration. Subject GL0096 is a 63 year old FPLD2 patient (heterozygous for mutation *LMNA *R482Q) whose scan shows total sc fat loss in the calves, major sc fat loss in the mid-thigh and marbled appearance of muscle tissue due to severe amounts of fat being stored within the muscle.

Mid-calf and mid-thigh sections were positioned based on reference anatomical features. The mid-point of the tibia was selected for "mid-calf" measurements and the mid-point of the femur was selected for "mid-thigh" measurements (Figure 1). For both the mid-calf and mid-thigh regions we acquired image stacks comprised of 17 transaxial slice images of 3 mm thickness each, together comprising a total superior/inferior coverage of 51 mm. The MRI provided a bright, high-threshold adipose tissue signal in raw image data, relative to other tissues and background. Other tissues, such as muscle and connective tissue, appeared as dark regions with low threshold values in MR images (Figure 2). The whole body scans (Figure 1) are a composite of four mid-slice coronal stack images acquired at four stations (head/neck, thoracic/abdominal, pelvic/thigh, lower leg).

### Fat quantification method from MRI

**Figure 3:**
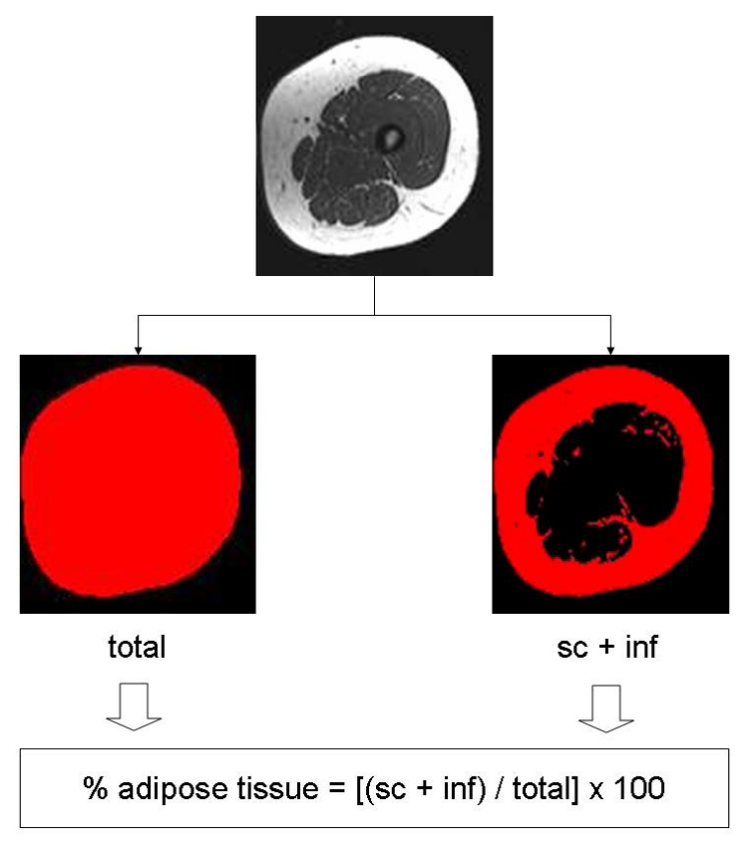
**Quantification of percent adipose tissue**. For each of the 17 transaxial slices in a given anatomical section, both the total volume and the total subcutaneous (sc) and connected infiltrated (inf) fat volumes were selected using the Connected Threshold Grower tool. Their corresponding volumes were determined using the Voxel Counter tool. The percent adipose tissue was calculated for each slice by dividing the total voxels determined for the sc + inf fat by the total voxels for the slice. The percent adipose tissue was determined for each slice alone and also for the overall section, combining the results from all 17 slices.

Analysis of the MRI stack images and measurements of subcutaneous adipose tissue was done by a single observer using analysis protocols developed in our laboratories (Figure 3). For each MRI data set acquired, the subcutaneous adipose tissue volume was quantified using ImageJ version 1.34 n image analysis software [9], specifically utilizing the Connected Threshold Grower and Voxel Counter tools. Subcutaneous adipose tissue was defined as the adipose tissue that circulated the circumference of the lower limb, adjacent to the skin, as well as any connected adipose tissue that was infiltrated into the muscle.

Prior to image analysis and fat quantification, all raw images underwent a preprocessing stage using the auto-brightness tool in order to minimize background noise and improve the quality of the images as much as possible. Images were further standardized with a distance in pixels set at 1.00 pixel/mm and the image dynamic was reduced to an 8-bit type to match the requirements of the used software. Starting from a user-defined seed point within the subcutaneous adipose tissue in the image, the method utilized the Connected Threshold Grower tool to create a connectedness map of the volumetric contours of subcutaneous adipose tissue within the image stack. This map represented the strength of connectivity between the seed point in the subcutaneous region and every voxel (volumetric pixel) in the image stack. Total tissue and adipose tissue threshold value ranges were obtained by manually sampling the signal intensity in each image stack. Using this threshold selection mechanism, the connectedness map of subcutaneous adipose tissue was then thresholded to segment the volume to be analyzed. Finally, the contours of segmented subcutaneous adipose tissue were quantitated using the Voxel Counter tool, which produced the final output voxel count within the volumetric region.

Percent adipose tissue was calculated by dividing the total voxels determined for fat intensity signals connected to the subcutaneous adipose seed point by the total voxels for the slice (Figure 3). After the percent adipose tissue was calculated for each slice, the average of 17 slices was reported as the average percent adipose tissue/slice. The percent volume of fat for each region was also obtained by adding the fat intensity voxels for all 17 slices (total fat volume) and then dividing them by the sum of the total voxel areas (total region volume).

### Statistical analysis

Subcutaneous adipose measurements from MRI were statistically analyzed using SAS version 8.2 (SAS Institute, Cary, NC). The Pearson correlation coefficient was used to test variation among duplicate blinded subcutaneous adipose tissue measurements made by the same observer on different days in the mid-calf and mid-thigh (intra-observer variability) and also to test inter-observer variation for the same samples, analyzed by a different observer. The t-test for unequal variances was used to test for differences in mean percent subcutaneous adipose between FPLD subjects and normal controls. A nominal *P*-value < 0.05 was chosen as the threshold for significance for all statistical comparisons.

## Results

### Baseline clinical and anthropometric features of study subjects

The clinical and anthropometric features of the study subjects are displayed in Table 1. Compared to control subject GL2784 (female, aged 24), control subject GL2990 (female, aged 50), who met blood pressure and waist circumference cut points of the National Cholesterol Education Program Adult Treatment Program III (NCEP ATP III) criteria for diagnosis of the metabolic syndrome (MetS), had higher BMI, waist circumference (but not ratio of waist-to-hip circumference), and percent body fat (PBF) for the total body and lower extremities, as measured by segmental body composition MRI analysis. Compared to controls, FPLD3 subject GL0658, who also met the blood pressure and waist circumference cut points of the NCEP ATP III criteria for MetS, had similar ratio of waist-to-hip circumference, similar BMI and percent lower extremity fat to age-matched control GL2990, but lower total body PBF compared to the same age-matched control (Table 1). Compared to controls, FPLD2 subject GL0096, who also met blood pressure and waist circumference criteria of the NCEP ATP III definition for MetS, had increased ratio of waist-to-hip circumference (android pattern). BMI and PBF for the total body and lower extremities for FPLD2 subject GL0096 was similar to young control GL2784 and significantly less than older control GL2990 (Table 1).

### Qualitative differences on survey MRIs

Qualitative coronal regional fat distribution profile differences between affected and normal controls are shown in Figure 1. The main visible differences included: 1) greater subcutaneous fat depots, especially around the hips and thighs, for the two control subjects compared with the FPLD3 and FPLD2 subjects; and 2) attenuation of subcutaneous fat stores at a lower point on the thigh of the FPLD3 subject compared to the FPLD2 subject.

### Intra- and inter-observer correlations for quantitative MRI analysis

Intra-observer correlation was determined by comparing two replicates of percent subcutaneous fat for both the mid-calf and mid-thigh derived from subjects GL2784, GL2990, GL0658 and GL0096. Each replicate involved analysis of 17 transaxial images for both the right and left sides. Intra-observer correlation coefficients based on at least 68 sections each were, on average, 0.996 for the mid-calf and 0.998 for the mid-thigh. Inter-observer correlation was determined by comparing percent subcutaneous fat for both the mid-calf and mid-thigh derived from subjects GL2784, GL2990, GL0658 and GL0096, as measured by two independent observers. Each determination involved analysis of 17 transaxial images for both the right and left sides. The overall inter-observer correlation coefficients were, on average, 0.988 for the mid-calf and 0.991 for the mid-thigh.

### Quantification of subcutaneous fat from MRI

Quantification of the percent subcutaneous adipose tissue present in the mid-calf and mid-thigh regions showed that the control subjects had values ranging from 26-56%, with mid-thigh values always greater than mid-calf values. The older control subject GL2990 (BMI 34.8) had percent subcutaneous adipose tissue values that were ~1.3-fold greater than the younger, normal weight control subject GL2784 *P *< 0.0001). The FPLD3 subject GL0658 had significantly lower percent adipose tissue values for both the mid-calf and mid-thigh regions in comparison to both control subjects (*P *< 0.0001 for both). The most significant attenuation in subcutaneous adipose tissue was observed for the FPLD2 subject, where no subcutaneous connectedness map of fat was attainable for the minute remnants of adipose tissue present in the perimeter of mid-calf region, and thus quantification using the automated Connected Threshold Grower tool was impossible. The percent subcutaneous adipose tissue in the mid-thigh of FPLD2 subject GL0096 was also significantly lower than that observed for FPLD3 subject GL0658 (24.3 ± 3.7% *vs *34.4 ± 2.5%, *P *< 0.0001).

Mean and overall percent adipose tissue values are reported (Table 1), representing averaged values of individual slices composing each respective image stack and values of total volume subcutaneous adipose tissue respectively. Each of these values is also an average of two replicate data sets from two independent analyses. The correlation (r) between mean subcutaneous fat areas and overall fat volume was 0.99998.

## Discussion

Using a strategy to quantify subcutaneous fat in the lower extremity that was based on connectivity analysis, we found significant differences between subcutaneous adipose tissue in the mid-calf and mid-thigh sections of FPLD patients compared to normal controls. We found significantly reduced lower extremity subcutaneous adipose tissue in a subject with FPLD2 than in a subject with FPLD3. Specifically, no subcutaneous adipose tissue could be quantified in the calf of the FPLD2 patient compared to 19.2 ± 1.7% subcutaneous adipose tissue in FPLD3 (*P *< 0.0001). Similarly, the percent subcutaneous adipose tissue in the thigh was 24.3 ± 3.7% and 34.4 ± 2.5% (*P *< 0.0001), for the FPLD2 and FPLD3 patients respectively.

Current clinical assessment of adipose tissue distribution in common obesity and metabolic syndrome and subjects with FPLD2 and FPLD3 is still in its infancy. Also, BIA failed to capture differences in percent fat in lower extremities in FPLD2 *vs *FPLD3 perhaps because so much fat was infiltrated into muscle in FPLD2. In contrast, MRI adipose connectedness maps and semi-automated subcutaneous adipose tissue quantification with very high resolution and reproducibility, captured traits that could be compared statistically, confirming the subtle clinical differences [3,10].

This semi-automated method involved a Connected Threshold Grower tool which specified inclusion of only adipose tissue connected to the initial subcutaneous seed point. Based on this pilot study of FPLD patients, we observed very high intra- and inter-observer correlation values: *r *> 0.99 and >0.98, respectively. In addition to its reproducibility, the described method yields results quickly and accurately, with minimal user intervention. The method was limited by including only connected infiltrated adipose tissue. However, given the imprecise definition of subcutaneous adipose tissue in extremities, we elected to include the connected infiltrated adipose tissue in our calculations, again since this would require no user judgment and/or intervention, thus reducing another potential source of analytic variation. An additional limitation inherent in the ImageJ software, which does not affect reproducibility but affects image dynamic, is that of the 16-bit to 8-bit change to the image stacks prior to analysis. This reduction in image dynamic, which reduces resolution, is a common setback in medical image processing where similar general-purpose software libraries are used. Future development of the software to utilize original raw images would be advantageous in maintaining image integrity and reflecting more accurate analysis data acquired from quantification.

Evaluating FPLD patients theoretically allowed for assessment of the lower limits of resolution of the method; however, the method appeared insensitive for calf adipose measurements in FPLD2, since there was no subcutaneous fat according to the definition specified in the quantification methodology. Future application of this quantification method may include quantification of both thigh and calf depots for "garden variety" obesity, metabolic syndrome or diabetes. This approach might also be applicable to quantify metabolically important substrata of fat [11].

We recognize that this study was limited due to the small subject numbers from whom subcutaneous adipose tissue values were extracted. Acquisition of such values from a larger number of patients with both FPLD subtypes would verify the likely results observed here. Furthermore, controls were not ideally matched for age and BMI: while the FPLD2 patient had a similar BMI as the young control individual, unmeasured and uncontrolled factors related to age might have further contributed to variation in subcutaneous adipose tissue. Expanding the sample size in future studies would clearly be helpful in this regard.

The whole body scans suggested that this method can be adapted for other fat depots or bodily organs. However, widespread application would depend on development of standards with respect to regions surveyed, anatomical landmarks, number of measurements, etc – similar to the consensus standards agreed upon for carotid intima-media thickness measurements using ultrasound. Also, intramuscular fat is distributed either in intra- or inter-myocellular depots; which could be more specifically evaluated using proton magnetic resonance spectroscopy (MRS) and/or fat selective MRI [12-14]. Such regional distribution could be an additional MRI analyte that could be considered together with other intermediate traits in subjects with FPLD or even common metabolic syndrome. Furthermore, it is possible to obtain carbon-13 nuclear magnetic resonance (NMR) spectra of human muscle glycogen *in vivo *in diabetic patients [15], which has helped understand the pathogenesis of insulin resistance, metabolic syndrome and type 2 diabetes. Quantification of fat depots using MRI and appropriate image analysis software could provide complementary analytes for research and perhaps eventually for the diagnosis and monitoring of interventions.

## Conclusion

In summary, we report the use of MRI and image analysis software employing Connected Threshold Grower and Voxel Counter tools to help quantify lower extremity subcutaneous fat depots in patients with two molecular forms of partial lipodystrophy. We also showed that the measurements showed high intra- and inter-observer correlation in a small sample. Finally, the measurements could be compared statistically and thus confirmed the clinical impression that FPLD2 and FPLD3 differ with respect to the extent of subcutaneous fat loss; specifically, subcutaneous fat loss in the FPLD2 subject is greater than in the FPLD3 individual. Increasing the sample size of FPLD subjects in future studies will validate this interpretation. These tools can be applied immediately and might be useful in quantitative phenotype analysis of other forms of lipodystrophy and in less extreme disorders of fat redistribution or repartitioning, such as "garden variety" obesity, insulin resistance, or type 2 diabetes.

## Competing interests

The author(s) declare that they have no competing interests.

## Authors' contributions

SAA participated in the experimental design, data acquisition and analysis, interpretation of results, and manuscript writing. RLP participated in the analysis of the MRI data and manuscript writing. JFR participated in data acquisition, analysis and interpretation of results. BAM was involved in the clinical assessment. RW performed the MRI scans. BKR participated in the experimental design, data analysis, and interpretation of results. RAH participated in the experimental design, data analysis, interpretation of results and manuscript writing. All authors approved the final manuscript.

## Pre-publication history

The pre-publication history for this paper can be accessed here:



## Figures and Tables

**Table 1:** Characteristics of FPLD patients compared to controls

	**GL2784**	**GL2990**	**GL0658**	**GL0096**
Diagnosis	Control	Control	FPLD3	FPLD2
Mutation	Wild-type	Wild-type	*PPARG *F388L	*LMNA *R482Q
Age (years)	24	50	49	63
Sex	Female	Female	Female	Female
Height (m)	1.63	1.60	1.52	1.53
Weight (kg)	61.7	89.1	80.2	58.1
Body mass index (kg/m^2^)	23.5	34.8	33.4	24.8
Waist circumference (cm)	78.8	103.6	105.7	88.3
Waist-to-hip circumference ratio	0.88	0.86	0.88	0.92
Blood pressure (mmHg)	113/63	154/89	138/88 (treated)	131/76 (treated)
BIA measures (PBF, %)				
total body	28.0 ± 0.2	47.5 ± 0.2	31.8 ± 0.2	29.7 ± 0.1
right leg	31.4 ± 0.1	49.5 ± 0.1	44.0 ± 0.2	36.8 ± 0.1
left leg	31.3 ± 0.1	49.2 ± 0.1	47.9 ± 0.5	37.7 ± 0.1
Mean sc+inf volume/slice (%)				
mid-calf	26.3 ± 1.1	34.8 ± 1.5	19.2 ± 1.7	N/A
mid-thigh	44.3 ± 2.1	56.1 ± 1.5	34.4 ± 2.5	24.3 ± 3.7
Overall sc+inf volume (%)				
mid-calf	26.2	34.7	19.2	N/A
mid-thigh	44.5	56.1	34.5	24.5

Abbreviations: FPLD, familial partial lipodystrophy; BIA, bioimpedance analysis; PBF, percent body fat; MRI, magnetic resonance imaging; sc+inf fat, subcutaneous plus infiltrated fat; N/A, could not be assessed (no visible subcutaneous fat)

## References

[B1] Garg A (2004). Acquired and inherited lipodystrophies. N Engl J Med.

[B2] Hegele RA (2004). Phenomics, lipodystrophy, and the metabolic syndrome. Trends Cardiovasc Med.

[B3] Hegele RA (2005). Lessons from human mutations in PPARgamma. Int J Obes (Lond).

[B4] Agarwal AK, Garg A (2002). A novel heterozygous mutation in peroxisome proliferator-activated receptor-gamma gene in a patient with familial partial lipodystrophy. J Clin Endocrinol Metab.

[B5] Garg A, Peshock RM, Fleckenstein JL (1999). Adipose tissue distribution pattern in patients with familial partial lipodystrophy (Dunnigan variety). J Clin Endocrinol Metab.

[B6] Garg A, Vinaitheerthan M, Weatherall PT, Bowcock AM (2001). Phenotypic heterogeneity in patients with familial partial lipodystrophy (dunnigan variety) related to the site of missense mutations in lamin a/c gene. J Clin Endocrinol Metab.

[B7] Iacobellis G (2005). Imaging of visceral adipose tissue: an emerging diagnostic tool and therapeutic target. Curr Drug Targets Cardiovasc Haematol Disord.

[B8] Liou TH, Chan WP, Pan LC, Lin PW, Chou P, Chen CH (2006). Fully automated large-scale assessment of visceral and subcutaneous abdominal adipose tissue by magnetic resonance imaging. Int J Obes (Lond).

[B9] ImageJ: Image processing and analysis in Java. http://rsb.info.nih.gov/ij/.

[B10] Hegele RA, Cao H, Frankowski C, Mathews ST, Leff T (2002). PPARG F388L, a transactivation-deficient mutant, in familial partial lipodystrophy. Diabetes.

[B11] Smith SR, Lovejoy JC, Greenway F, Ryan D, deJonge L, de la Bretonne J, Volafova J, Bray GA (2001). Contributions of total body fat, abdominal subcutaneous adipose tissue compartments, and visceral adipose tissue to the metabolic complications of obesity. Metabolism.

[B12] Boesch C, Slotboom J, Hoppeler H, Kreis R (1997). In vivo determination of intra-myocellular lipids in human muscle by means of localized 1H-MR-spectroscopy. Magn Reson Med.

[B13] Brechtel K, Jacob S, Machann J, Hauer B, Nielsen M, Meissner HP, Matthaei S, Haering HU, Claussen CD, Schick F (2000). Acquired generalized lipoatrophy (AGL): highly selective MR lipid imaging and localized (1)H-MRS. J Magn Reson Imaging.

[B14] Schick F, Eismann B, Jung WI, Bongers H, Bunse M, Lutz O (1993). Comparison of localized proton NMR signals of skeletal muscle and fat tissue in vivo: two lipid compartments in muscle tissue. Magn Reson Med.

[B15] Petersen KF, Shulman GI (2002). Pathogenesis of skeletal muscle insulin resistance in type 2 diabetes mellitus. Am J Cardiol.

